# Hypercarnivorous apex predator could provide ecosystem services by dispersing seeds

**DOI:** 10.1038/srep19647

**Published:** 2016-01-21

**Authors:** José Hernán Sarasola, Juan Ignacio Zanón-Martínez, Andrea Silvina Costán, William J. Ripple

**Affiliations:** 1 Centro para el Estudio y Conservación de las Aves Rapaces en Argentina (CECARA), Facultad de Ciencias Exactas y Naturales, Universidad Nacional de La Pampa, Avda. Uruguay 151, 6300 Santa Rosa, La Pampa, Argentina; 2 Instituto de las Ciencias de la Tierra y Ambientales de La Pampa (INCITAP), Consejo Nacional de Investigaciones Científicas y Técnicas, Avda. Uruguay 151, 6300 Santa Rosa, La Pampa, Argentina; 3 Global Trophic Cascades Program, Department of Forest Ecosystems and Society, Oregon State University, Corvallis, OR 97331, USA

## Abstract

Large “hypercarnivorous” felids are recognized for their role as apex predators and hence as key elements in food webs and ecosystem functioning through competition and depredation. Here we show that cougars (*Puma concolor*), one of the largest and the most widely ranging apex felid predators with a strictly carnivorous diet, could also be effective secondary long distance seed dispersers, potentially establishing direct and non-herbivore mediated interactions with plant species at the bottom of the food web. Cougars accidently ingest and disseminate large amounts of seeds (31,678 seeds in 123 scats) of plant species initially consumed by their main prey, the Eared Dove *Zenaida auriculata*. The germination potential of seeds for the three plant species most abundantly found in cougar scats (19,570 seeds) was not significantly different from that observed in seeds obtained from dove gizzards, indicating that seed passage through cougar guts did not affect seed germination. Considering the estimated cougar density in our study area, dispersal of seeds by cougars could allow a mean, annual seed spread of ~5,000 seeds per km^2^. Our results demonstrate that strictly carnivorous, felid predators could have broad and overlooked ecological functions related to ecosystem structuring and functioning.

Seed dispersal is one of the most important mutualistic interactions between vertebrate animals and plants^1^ and is a particularly key process sustaining plant populations, primary productivity and natural community dynamics^2^. Secondary long distance dispersal of seeds, which implies the transport of plant propagules by more than one dispersal vector, allows seeds to reach and colonize areas far away from their parent plants, resulting in an important process in species demography and genetic structure, particularly in degraded and fragmented landscapes^3^. However, despite its biological significance, secondary dispersal of seeds remains a complex and little understood ecological process^4^.

Felids comprise a widespread family of mammals within the Order Carnivora that includes medium-sized wildcats to the largest representatives in this group, such as jaguars (*Panthera onca*), tigers (*Panthera tigris*) and lions (*Panthera leo*). Unlike other large carnivorous mammals, daily protein requirements restrict felids to a highly exclusive carnivore diet, gaining them the term of “hypercarnivores”^5^. The cougar (*Puma concolor*) has a global distribution from northern British Columbia in Canada across western North America and Central and South America to the southern tip of Chilean Patagonia , being the most widespread mammal in the Western Hemisphere^5^,^6^. As with other large apex predators, the ecological role of cougars in natural communities is mainly associated with predation and competition. The former includes predatory interactions that could affect the behaviour, demography and population dynamics of their prey, which may sometimes include other predators (i.e. intra-guild predation), whereas competition encompasses inter-specific interactions with other top predators located in the same trophic level. Indirect effects of apex carnivorous predators on primary producers in the lower trophic levels are expected only through “top-down” and “trophic cascade” effects^7^,^8^,^9^,^10^,^11^. By such mechanisms, predatory pressures that apex predators exert on their herbivore prey populations may indirectly diminish the effects of herbivores on primary producers, thus playing a key role in the structure and functioning of natural communities. Documented examples of trophic cascade process on natural communities promoted by cougars are those in which these predators were either reduced in number or completely removed from natural systems, mainly due to anthropogenic factors (persecution, habitat loss or fragmentation), thus relaxing their function of limiting herbivore densities^12^,^13^. However, direct, non-herbivore-mediated interactions between strictly carnivorous apex mammal predators and plants have not been established for any natural system.

Here we show that cougars may play a key role in plant community dynamics through effective secondary dispersal of seeds. This widespread apex predator is able to spread seeds ingested by their main, granivorous prey, the eared dove (*Zenaida auriculata*; hereafter, “doves”). This novel function for cougars could have direct impacts on the spread and establishment of new plant individuals with potential implications on the maintenance of gene flow between plant populations or on the colonization of new areas by native or exotic plant species.

## Results

### Predator diet and seed consumption

**Figure 1:**
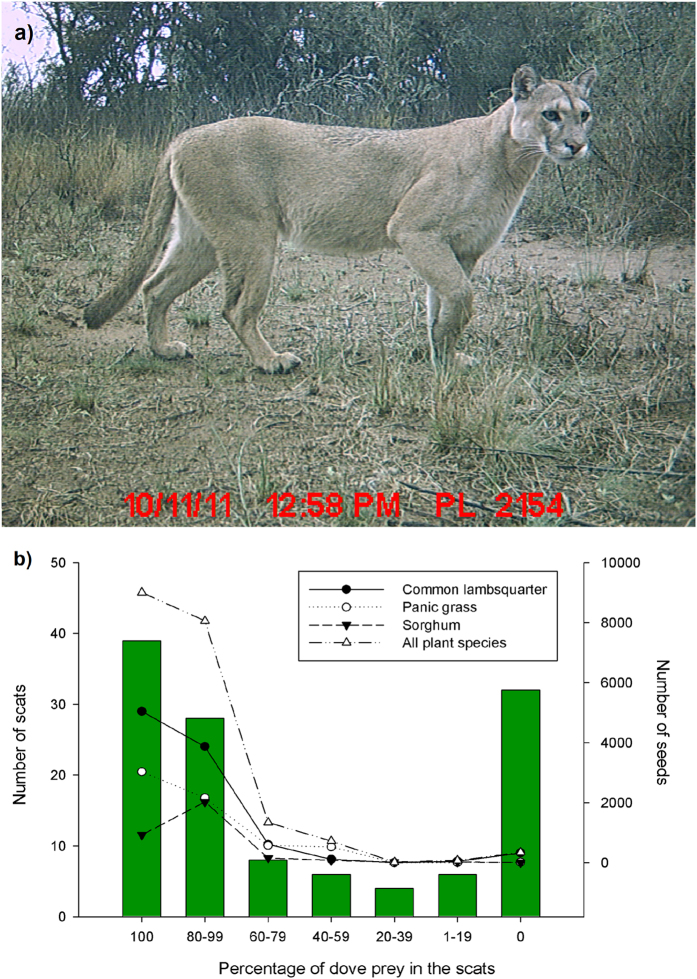
(**a**) Cougar photographed by camera traps in the study area and (**b**) relationship between total numbers of seeds for three plant species found in cougar scat and the percentage of eared doves remains in them visually estimated from dry and disaggregated scat contents.

We collected a total of 123 cougar scats through years 2010 to 2012 at the Parque Luro Natural Reserve, La Pampa province, central Argentina. We were able to collect cougar scats during all seasons including the following months: January-February (austral summer), June (austral autumn), July-August (austral winter) and November (austral spring). The cougar’s diet was composed mainly of doves (54% of prey items consumed) followed by small and medium sized mammals (19.2%), ungulates (red deer *Cervus elaphus* and wild boar *Sus scrofa*; 16.3%), and other small birds (5.8%). We counted a total of 31,678 seeds in cougar scats (mean 257.5 seeds per scat). From these seeds, three identified herbaceous species comprised the bulk of seeds in scats with 19,570 seeds: common lambsquarter (*Chenopodium album*), an exotic summer-annual weed species, panic grass (*Panicum bergii*), a perennial native grass, and sorghum (*Sorgum bicolor*), a cultivated grass (Table 1). We did not find the remains of grasses in scats that would indicate accidental seed consumption by cougars, such as during handling of prey. Furthermore, seeds occurred in a high percentage (85%, 104 scats) of the analysed scats, a level that likely precludes accidental seed consumption. Moreover, the occurrence of total seeds in scats was positively and significantly associated with the co-occurrence of doves in the scats (χ^2^ = 42.8, d.f. = 1, *P* < 0.01; Fig. 1). Only 347 of the total number of seeds (1%) were found in cougar scats that did not contain dove remains, although their occurrence in scats was presumably linked to previous consumption of doves by cougars.

### Germination potential of seeds

**Figure 2:**
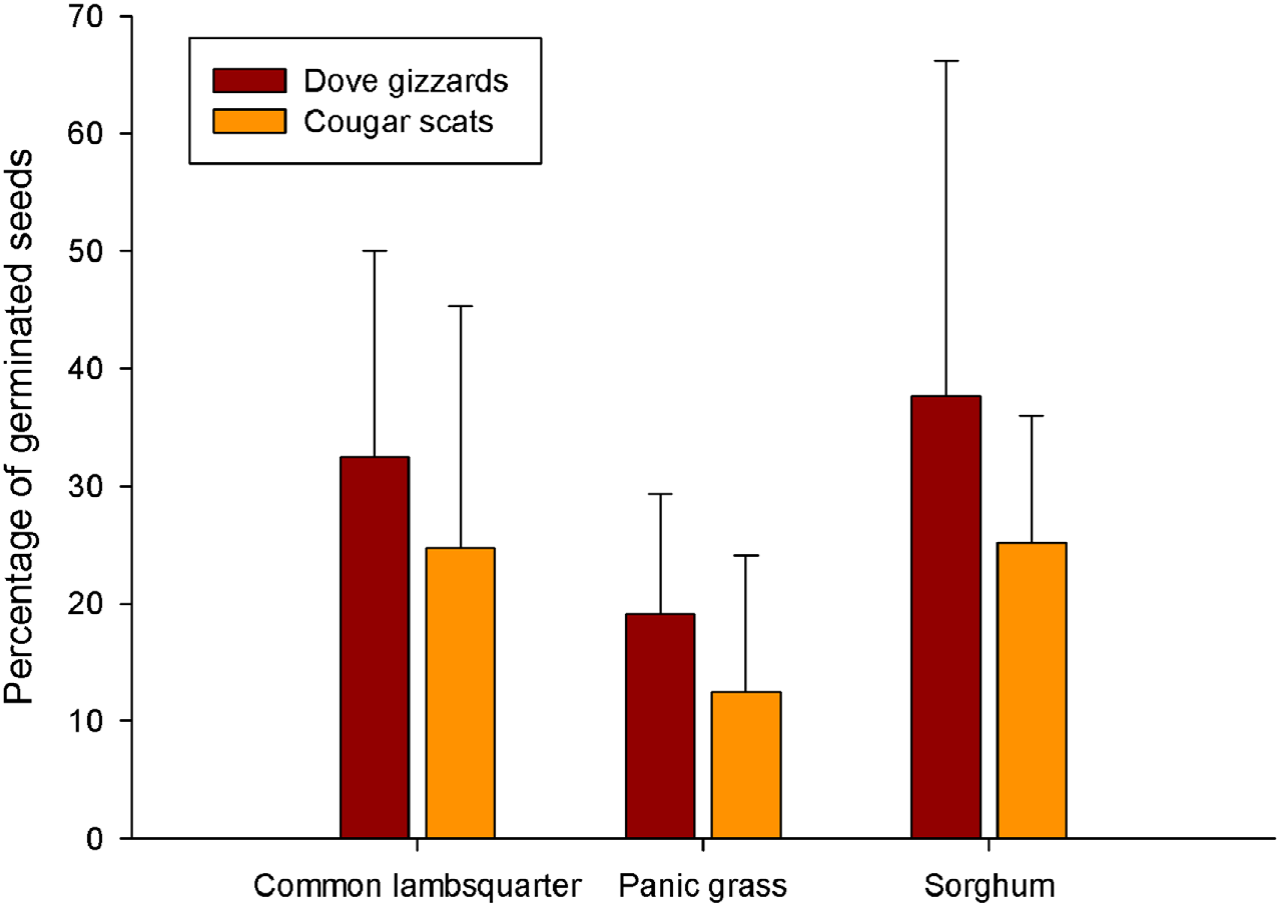
Mean (±SD) germination potential of seeds of three plant species at each of the two steps along the seed dispersal pathway (i.e. eared doves and cougars). Differences in germination potential between seeds obtained from dove gizzards and those collected from cougar scat were not significant (P > 0.25 in all cases).

We performed germination trials for these three more frequent plant species, considering both seeds from scats and from dove gizzards. Mean germination potential for seeds found in cougar scat (*N* = 382 seeds from 31 scats) was not significantly different from that obtained from dove gizzards (*N* = 359 seeds from 44 gizzards) for all three plant species (common lambsquarter: *t*_(9, 27)_ = 1.05, *P* = 0.30; panic grass: *t*_(5, 7)_ = 1.11, *P* = 0.29; sorghum: *t*_(14, 7)_ = 1.18, *P* = 0.25; Fig. 2), indicating that seed passage through cougars did not affect the viability and germination potential of seeds.

### Cougar’s density and seed dissemination

The estimated density (±SE) of adult cougars in the Natural Reserve and surrounding buffer area around camera trap-grid was 8.8 (±1.95) individuals per 100 km^2^ (Zanón-Martínez *et al.*, unpublished data). Test statistics provided little evidence that this cougar population violated the assumption of closure during our surveys (*P* = 0.11). The capture–recapture analysis showed that cougar capture probability per sampling occasion estimated under the best recapture model (capture probability = 0.25) was constant (Zanón-Martínez *et al.*, unpublished data).

**Figure 3:**
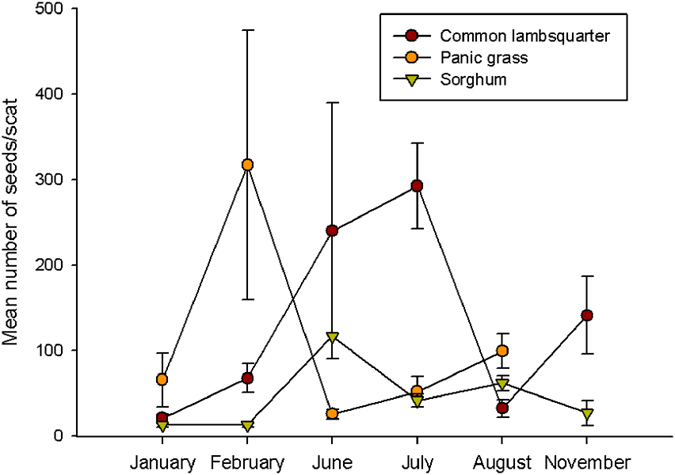
Field temporal deposition of seeds through secondary seed dispersal by cougars. Values of seeds in cougar scats collected in the study area are expressed as the mean number of seeds/scat (±SE) in each of field sampling occasions.

With the exception of panic grass, which was not present in scats collected during spring, seeds of these plant species variably occurred in cougar scats throughout the year irrespective of the plant fruiting cycle (Fig. 3). Considering cougar density at the site and the occurrence of seeds in scats for only these three most common grass species, we estimate that cougars were able to disseminate as much as ~5,000 seeds per km^2^ per year (Table 1).

## Discussion

Our results show that large apex hypercarnivorous felids may potentially and directly interact with primary producers at the bottom of food webs by processes other than by limiting herbivore populations and subsequent trophic cascades, but with similar potential for additional ecosystem effects^11^. In addition to the potential local demographic effects on plant populations for the recruitment of new individuals, long distance seed dispersal involving cougars may play a role in large-scale ecosystems processes such as the maintenance of metapopulation dynamics, gene flow between populations and the colonization of new and unoccupied habitats by plants^4^. While the estimation of demographic effects on plant populations could be affected by the estimated number of dispersing individuals (i.e. abundance of cougars) that later determines the final number of seeds disseminated, processes of connectivity, gene flow and colonization of new areas are usually determined by the dispersion and establishing of only a small number of new seedlings per year. However, the extent and quantification of any ecosystem effects due to cougars preying on seed-eating birds should be determined through additional research.

Long distance seed dispersal process involving top bird predators as secondary disperser are well documented and studied in depth only in the Canary Archipelago^14^,^15^,^16^. In this volcanic island ecosystems, two predatory birds, the Eurasian kestrel (*Falco tinnunculus*) and the southern grey shrike (*Lanius meridionalis*), are effective secondary dispersers of a variety of seeds of fleshy-fruited plants previously consumed by one of their main prey, the frugivorous lizards of genus *Gallotia*. These seed dispersal systems involving top-predators, frugivorous prey and plants could be important for the spread and distribution of plant species among these islands, particularly in volcanic zones were lava flows create young substrates for potential plant colonization^15^.

The process of long-distance seed dispersal may also be of conservation concern because it could facilitate the spread of invasive plant species^3^. Long distance dispersal of seeds by cougars could also promote range expansion of alien plant species. The most important plant species in our study in terms of the number of seeds dispersed was the common lambsquarter, a weed native from Europe and Asia but currently cosmopolitan and widely distributed across temperate and tropical regions of the world.

Doves are not able to disperse viable seeds because all the seeds they ingest are destroyed by their powerful gizzards^17^. However, cougar predation on doves partially counteracts previous seed removal by birds, allowing the dispersal of seeds that otherwise are eliminated from plant populations as a consequence of granivory by birds. In addition, seed dispersal from predator-prey interaction of these two generalized dispersal vectors may result in relatively high levels of dispersal of seeds, due to both the high mobility and seed carrying capacity of each of them. Doves may carry thousands of seeds in their crops and are able to travel up to 120 km daily from breeding colonies to foraging sites^17^, whereas cougars move up to four km in just five hours between camera trap stations in our study area.

Unlike other documented processes of primary and secondary seed dispersal by endozoochory, where seed dispersal takes place in synchrony with plant reproductive phenology after plant fruiting, the occurrence of seeds in cougar scats was distributed throughout the year (Fig. 3). This singularity in the temporal pattern of secondary dispersal process by cougars is largely determined by the foraging strategies of the eared doves, which are able to consume seeds from mother plants but also from seeds available all year round in the seed bank (Costan and Sarasola, unpublished data).

Processes of secondary dispersal of seeds as those described here may be widespread, since birds as a group are the most important seed-predators in most ecosystems and overlap with wild felids in every biome and habitat where the latter are present. In the particular case of doves and cougars, population outbreaks of this bird species have also been registered in many countries throughout South America, with breeding colonies of several millions of birds reported in Argentina and Uruguay and in tropical regions of Colombia, Bolivia and Brazil^18^ where diversity and abundance of large and medium sized felids is high^5^,^6^. In our study area at the Parque Luro Natural Reserve, for example, abundance of doves has been estimated at over 3 million individuals (authors’ unpublished data). The eared dove population in the United States is estimated at about 274 million individuals^19^, and other gregarious and granivorous dove species, such as the white-winged dove (*Zenaida asiatica*) may also reach high densities in western United States and Mexico^20^. Although, cougars are recognized as generalist predators that forage on the most abundant available prey at each site, most of the studies on the diet of cougars through the Americas document cougar preference for large ungulates. However, there are considerable geographical gaps regarding the knowledge of cougar’ food habits, particularly for tropical areas in the Neotropical regions. Furthermore, cougars’ opportunistic consumption of highly gregarious and abundant birds have been previously reported. In Monte Leon National Park in southern Argentinian Patagonia, for example, the Magellanic penguin (*Spheniscus magellanicus*) was the most important native prey item in the diet of cougars in terms of occurrence (38.2% of Magellanic penguins vs. 25.5% of native ungulates) and second in importance among native prey in terms of biomass (24.3%)^21^.

Wild felids face a variety of threats that have caused substantial declines in their populations and contractions of their geographic ranges^11^,^22^. Cougars are not an exception and their range has suffered significant contractions due to human pressure during the last two centuries^23^. Fortunately, there is increasing recognition that removing predators from natural ecosystems comes with severe consequences, especially considering those effects derived from the lack of top-down control on the food webs and resulting cascade effects on biodiversity^7^,^22^,^24^. Other overlooked, little studied or still unknown ecosystem functions of feline species may also disappear along with their populations.

## Methods

We conducted a cougar food habit analysis from scats collected at the Parque Luro Natural Reserve (7600 ha), La Pampa Province, Argentina. The Reserve is surrounded by a mosaic of semiarid forest remains and agriculture lands. It is dominated by caldén tree (*Prosopis caldenia*) forests with the characteristic savannah-like landscape typical of the Espinal Biome^25^. Cougar scats were collected throughout the year between 2010 and 2012 along approximately 120 km of internal and peripheral roads available at the Reserve. Once in the lab, scats were hydrated to allow prey remains and seeds separation. Prey remains were identified using reference collections for birds and other prey and keys for mammals^26^. Cougars’ diet composition was expressed as percentage of frequency (i.e. the number of times an item appears in the diet over the total number of prey items in the sample). Seeds of plant species were identified using available keys^27^. We employed chi-square test with Yates continuity correction^28^ to examine association between co-occurrence of doves and seeds on cougar scat.

With the objective of comparing the germination potential of seeds from the different seed dispersal pathways, we also obtained seed samples from dove gizzards for these three plant species by capturing doves using mist nets and inducing regurgitation by the birds through providing them with an emetic solution of 1.5% tartrate antimony potassium^29^. Birds caught were weighed and the emetic formula administrated at a dosage of 0.8 ml of solution per 100 g body weight^29^. Once supplied with the emetic solution, birds were placed in individual boxes (30 × 30 × 20 cm) and kept there for a maximum period of 60 min. After that time the birds were released and the regurgitated content of their crops collected from the box’s door and stored in paper envelopes until their analysis in the laboratory.

Germination potential of regurgitated seeds and seeds recovered from cougar scat was tested by sowing them individually in pots with a soil substrate from the study site. Pots were watered regularly and kept in a greenhouse at 25 °C. We checked for germination (i.e. sprout emergence) three times a week during three months until no new seeds germinated. Seeds belonging from each dove gizzard and scat were treated separately during germination tests and the percentage of seeds germinated was estimated from each sample. Parametric student *t*-tests were performed to examine differences in germination potential between seeds of the same plant species obtained from dove gizzards and cougar scats.

Cougar density in the Reserve was determined by deploying sixteen trapping stations composed of two camera-traps (Moultrie Game Spy 4.0 MP DGTL, Moultrie Products, LLC., Alabaster, Alabama) placed opposite each other on both sides of available trails or roads. This methodology has been successfully employed to estimate cougar densities^30^. Trapping stations were set at regular intervals (2–3 km) in a grid across the area and kept activated during 38 continuous days. Individual identification of cougars was made according to the protocol proposed in previous studies^30^. We established a cougar’s capture–recapture history by individual identification in the pictures (Fig. 1). Three investigators independently identified the cougar record on the photograph from camera traps to enumerate individual cougar that were identified by obvious and subtle markings (e.g., kinked tails, scars, ear nicks, tail-tip coloration and shape, or undercoat spot patterns). Disagreements by researchers over cougar IDs were settled by consensus to reach a mutual agreement or they were tossed out if agreement could not be reached, constructing a unique capture history for known individuals for the study site^30^. These capture-recapture histories were then analyzed using program CAPTURE^31^,^32^ to compute test statistics for the hypothesis of a closed population and model selection statistics based on a discriminant function developed from extensive simulations and to derive estimates of capture probability (*p*) and cougar abundance (*N*) at the study site. To determine the size of the area surveyed, the mean of the maximum distance moved between cameras was calculated for each cougar capture history and half this distance, and this value was used as the buffer radius around each camera trap location^30^,^33^,^34^. The number of cougars from the survey was then divided by the total survey area to obtain density of cougars and standard error estimated using the delta method^35^.

All field procedures and experimental protocols were approved by the Subsecretaría de Ecología and Dirección de Recursos Naturales del Gobierno de La Pampa (La Pampa province) in accordance with the provincial law number 1194 of La Pampa province (Argentina).

## Additional Information

**How to cite this article**: Hernán Sarasola, J. *et al.* Hypercarnivorous apex predator could provide ecosystem services by dispersing seeds. *Sci. Rep.* **6**, 19647; doi: 10.1038/srep19647 (2016).

## Figures and Tables

**Table 1:** Estimates of mean annual seed field deposition (±SE) for three herbaceous species as a result of secondary dispersal of seeds by cougars.

Plant species	Seeds in scats	Seeds per scat a	Total seed deposition^b^
Common Lambsquarter	10,006	81.35 (±16.18)	2,613 (±520)
Panic grass	6,318	51.37 (±12.34)	1,650 (±396)
Sorghum	3,246	26.39 (±4.29)	848 (±138)
Total	19,570		5,110 (±1,054)

^a^Mean number of seeds in scats (*N* = 123 scats).

^b^Seeds per scat × 0.088 cougars/km^2^ × 365 days, with defecation rate taken to be one scat per cougar per day.
